# 手术诱发获得性凝血因子Ⅴ抑制物并发致命性出血1例报告并文献复习

**DOI:** 10.3760/cma.j.cn121090-20241128-00491

**Published:** 2024-12

**Authors:** 贇和 张, 娴 潘, 小雪 楚, 玮 徐

**Affiliations:** 上海市东方医院重症医学科，上海 200120 Department of Critical Care Medicine, Shanghai East Hospital, Shanghai 200120, China

**Keywords:** 因子Ⅴ, 凝血因子抑制物, 糖皮质激素, 免疫抑制法, 血浆置换, Factor V, Blood coagulation factor inhibitors, Glucocorticoids, Immunosuppression, Plasma exchange

## Abstract

**目的:**

探讨获得性凝血因子Ⅴ抑制物患者的诊治方法及预后。

**方法:**

对2022年上海市东方医院重症医学科收治的1例获得性凝血因子Ⅴ抑制物患者进行分析。并以［（"Factor Ⅴ" OR "Factor Ⅴ Inhibitors"）AND（"Autoantibodies" OR "Immune System Diseases"）］AND［acquired（Title/Abstract）OR autoimmune（Title/Abstract）OR inhibitor（Title/Abstract）］为检索式在PubMed数据库进行检索，以（获得性第五因子抑制物OR获得性凝血因子Ⅴ抑制物OR凝血因子Ⅴ抗体OR凝血因子Ⅴ自身抗体）AND（缺乏OR抑制OR功能障碍）为检索式在中文数据库（万方、维普、中国知网）中进行检索并文献复习。

**结果:**

患者为56岁男性，因术后凝血功能紊乱伴腹腔出血转入我科。辅助检查示血常规正常，凝血功能示凝血酶原时间（PT）76.7 s，活化部分凝血活酶时间（APTT）83.1 s，国际标准化比值（INR）7.14。凝血因子活性示Ⅴ因子8.4％且呈持续下降。混合血浆纠正试验示即刻和延迟2 h均无法纠正。凝血因子抗体滴度示Ⅴ因子抑制物8 BU/ml。考虑因手术创伤诱发获得性凝血因子Ⅴ抑制物生成，并导致致命性出血。予激素、环磷酰胺并血浆置换治疗后凝血异常纠正。文献检索共检索到国内报道16例，全球累计报道近200例。

**结论:**

获得性凝血因子Ⅴ抑制物患者临床表现多样，现有治疗手段包括支持治疗、糖皮质激素、免疫抑制剂、血浆置换及联合治疗，患者预后取决于诱发因素及诊治的时效性。

## Abstract (en)

**Objective:**

To explore the diagnosis, treatment, and prognosis of patients with acquired factor Ⅴ inhibitors.

**Methods:**

We analyzed a case of acquired factor Ⅴ inhibitor treated in the department of critical care medicine at Shanghai East Hospital in 2022. A literature review was conducted using the search terms［（"Factor V" OR "Factor V Inhibitors"）AND（"Autoantibodies" OR "Immune System Diseases"）］AND［acquired（Title/Abstract）OR autoimmune（Title/Abstract）OR inhibitor（Title/Abstract）］in the PubMed database. Chinese databases（Wanfang, VIP, and CNKI）were searched using（acquired factor V inhibitor OR acquired coagulation factor V inhibitor OR coagulation factor V antibody OR coagulation factor V autoantibody）AND（deficiency OR inhibition OR dysfunction）.

**Results:**

A 56-year-old male was admitted to the department of critical care medicine due to coagulopathy following surgery. Laboratory tests showed normal blood routine but revealed prolonged PT of 76.7 s, APTT of 83.1 s, and an INR of 7.14. Coagulation factor assays indicated factor V activity at 8.4％, which continued to decline. Immediate and 2-hour delayed mixing studies failed to correct the coagulation abnormalities. The factor V inhibitor titer was measured at 8 BU/ml. It was considered that surgical trauma induced the production of an acquired factor V inhibitor, leading to fatal bleeding. After treatment with corticosteroids, cyclophosphamide, and plasma exchange, the coagulation abnormalities were corrected. The literature search identified 16 cases reported in China and approximately 200 cases reported globally.

**Conclusion:**

Patients with acquired factor V inhibitors exhibit diverse clinical manifestations. Current treatment options include combined therapies such as supportive care, corticosteroids, immunosuppressants, and plasma exchange. The prognosis of patients depends on the inducing factors and the timeliness of diagnosis and treatment.

获得性凝血因子Ⅴ抑制物（acquired factor Ⅴ inhibitor, AFVI）是较为罕见的凝血系统疾病，其发病率估计为每百万人年<0.5[1]。AFVI的临床表现呈多样化，可见于无症状患者，或与血栓相关，也可导致危及生命的大出血。AFVI是一种针对凝血因子Ⅴ（Factor Ⅴ, FⅤ）轻链的IgG抗体，其临床表现的异质性源于抗体影响的靶表位不同，进而影响促凝抗凝平衡。由于其症状不典型，多数患者常就诊于非血液科，进一步增加了诊断难度。本文报道1例外科术后诱发获得性凝血因子Ⅴ抑制物并发致命性出血患者，对其临床特征、辅助检查、诊疗过程和疾病转归进行介绍，并对相关文献进行复习。

## 病例资料

患者男性，56岁，因右上腹痛1 d入院。患者既往体健，此次进食油腻食物后起病，诊断为胆囊结石伴急性胆囊炎。患者既往糖尿病病史5年，药物控制可。无手术史、无特殊疾病家族史。查体示腹略胀，右上腹压痛，全身皮肤黏膜无出血点，无黑便。入院后予禁食、头孢美唑抗感染治疗后患者症状明显缓解。1周后复查血常规：WBC 16.7×10^9^/L，HGB 134 g/L，PLT 268×10^9^/L，肝肾功能及凝血功能均在正常范围，外科遂行腹腔镜胆囊切除术。患者术后并发胆漏，反复发热腹痛。术后2周，患者突发剧烈腹痛伴血红蛋白下降，完善腹部CT示肝周及盆腔积液，考虑腹腔出血，急诊行剖腹探查术。术中见胆囊窝及肝周大量积血，总量约4 000 ml，手术当日输注红细胞7 U，血浆800 ml，冷沉淀10 U。患者术后转入重症医学科，予抗感染、保肝、营养支持等治疗后生命体征平稳，查血常规示：WBC 14.3×10^9^/L，HGB 118 g/L，PLT 177×10^9^/L，查肝功能示：总胆红素63.3 µmol/L，直接胆红素60.7 µmol/L，ALT 23 U/L，AST 11 U/L。但患者出现凝血功能显著异常，查血栓弹力图示：R值58.1 min，K值17.4 min，α角16.5°，MA值27.5 mm，EPL值1.05％，LY30 0.15％；凝血功能示：PT 76.7 s，APTT 83.1 s，国际标准化比值7.14，D-二聚体27.9 mg/L。病程中患者再次出现腹腔大出血1次，予剖腹探查术止血，术中见原创面弥漫性渗血，未见血管性出血，总出血量约1 500 ml。考虑凝血功能障碍不能用肝脏损伤解释，予凝血因子活性测定：Ⅱ因子21.4％，Ⅴ因子8.4％，Ⅶ因子28.5％，Ⅷ因子88.7％，Ⅸ因子63.8％，Ⅹ因子16.5％，Ⅺ因子52.2％，Ⅻ因子29.5％；PT和APTT纠正实验示：即刻和延迟2 h后均不可纠正，考虑存在共同途径凝血因子抑制物。外送凝血因子抗体检测示：Ⅴ因子活性1.2％，Ⅴ因子抑制物8 BU/ml。至此，该患者获得性凝血因子Ⅴ抑制物诊断明确。予甲泼尼龙60 mg/d联合环磷酰胺200 mg隔日1次治疗，同时立即予血浆置换1次，置换量为1.2倍血浆容量，约3 000 ml。治疗后患者凝血功能明显改善，但随后出现小幅反跳（图1）。遂于首次血浆置换3 d后及1周后各追加血浆置换1次，由于血浆获得受限，后两次置换量均为1倍血浆容量，约2 500 ml。治疗后患者生命体征平稳，复查凝血功能示：PT 44.9 s，APTT 58.6 s，INR 4.24。停用环磷酰胺并改为甲泼尼龙口服后转回普通病房，后甲泼尼龙逐渐减量直至停用。患者于1个月后顺利出院，出院时PT及APTT均已恢复正常，患者拒绝再次复查Ⅴ因子抑制物滴度。随访至出院后3个月，复查凝血功能均在正常范围内。

**图1:**
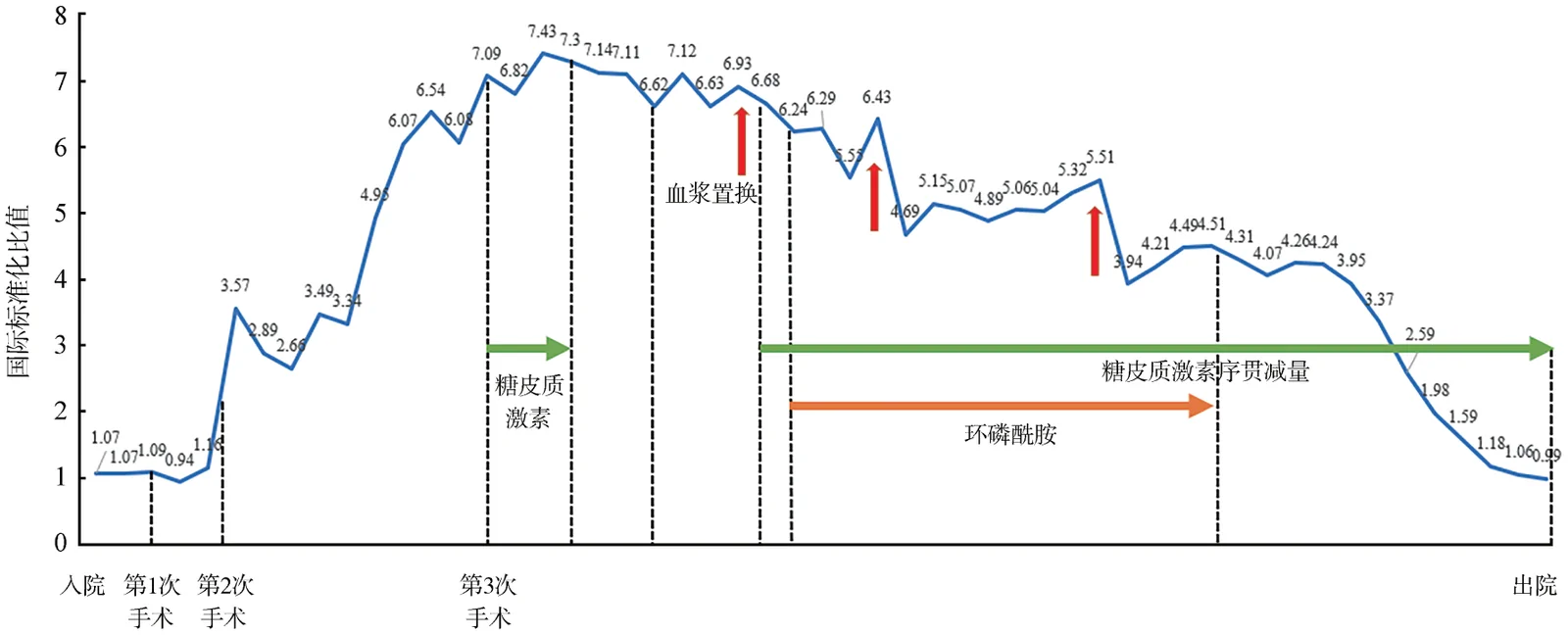
患者国际标准化比值变化趋势及主要治疗方案

## 讨论及文献复习

AFVI是指体内生成针对FⅤ的抑制物，影响FⅤ的活性，从而导致出凝血障碍。AFVI的临床表现缺乏特异性[2]。有研究表明，约20％患者无凝血系统临床表现，常在其他疾病就诊过程中偶然诊断[3]。在存在临床症状的患者中，最常见的表现是黏膜出血，其次是皮下出血和鼻衄。致命性出血如颅内出血或腹腔大出血的发生率为5％～10％[4]。患者常为单发病例，不伴凝血系统异常的个人史或家族史。

现有文献中报道的AFVI病例多与如下危险因素有关，包括暴露于牛源性凝血酶制剂、手术、抗生素的使用、输血、肿瘤、自身免疫性疾病等。早期报道大多集中于牛源性凝血酶制剂的暴露，牛源性Ⅴ因子具有极强的免疫源性，可诱导抗牛FⅤ抑制物的产生，进而与人FⅤ发生交叉反应[5]。然而近年来，包括我国在内的大部分国家已采用人类血浆制备凝血酶制剂，极大地减少了相关风险。本例患者考虑为手术创伤诱发抑制物产生，并可能在后续反复输血、抗感染过程中促进抑制物滴度升高。

与其他凝血因子抑制物不同，AFVI的滴度并不与临床症状的严重程度呈线性相关。有研究发现，出血和不出血的AFVI患者，其抑制物滴度的中位数均为19 BU，其原因可能为血小板结合FⅤ的活性部分保留以及AFVI识别表位的不同[6]。80％的FⅤ分布于血浆，此外有20％的FⅤ储存于血小板的α颗粒中。这部分血小板来源的FⅤ能发挥一定止血作用。一部分先天性FⅤ缺乏的患者即使血浆中FⅤ水平低到不可被检测，也很少发生严重出血事件，可能是因为残余的血小板结合FⅤ能够产生足量的凝血酶以预防出血。因此，AFVI是否引发出血可能取决于其是否影响血小板结合FⅤ，当AFVI合并血小板减少时将增加出血倾向[7]。此外AFVI识别结合的表位不同也会造成不同的临床表现。有研究表明，识别FⅤ轻链C2结构域的AFVI与出血表现相关。AFVI的识别通常源于PT和APTT的显著延长，这种延长不能为混合血浆纠正试验所纠正（包括即刻及延迟两个时相）。AFVI的确诊和滴度测定依靠Bethesda法[8]。与其他凝血因子抑制物不同，在体外实验中Ⅴ因子抑制物能立即使FⅤ失活，而常见的FⅧ抑制物在体外通常需要1～2 h的共同孵育才能完全灭活FⅧ[9]。本例患者抑制物滴度为8 BU/ml，低于文献报道的中位滴度，患者的血小板除两次大出血后出现一过性降低外，基本维持在正常范围。但整体来看，患者的出血风险极高，验证了抑制物滴度与出血风险不呈线性相关，但无法用血小板结合FⅤ数量来解释，这一现象可能与AFVI的识别位点相关。

AFVI的治疗包括两部分：控制出血及清除体内抗体。无症状患者通常不需要治疗。对于有出血症状的患者，可以选择输注血制品，尤其是血小板，或输注凝血物质进行旁路治疗，但具体疗效不明确[10]。血浆置换和免疫吸附可以迅速降低抑制物滴度。单独应用激素或联合环磷酰胺的免疫抑制治疗及大剂量免疫球蛋白冲击治疗也可能有效，但研究结果间存在矛盾[3],[11]。有研究报道了抗CD20单克隆抗体利妥昔单抗的治疗成功案例，常规治疗无效的患者也可考虑使用[12]。本例患者在明确诊断前已接受了包括血浆、凝血酶原复合物等在内的大量血制品输注治疗，但仍有明显出血倾向，考虑补充凝血物质的旁路治疗无效，予激素联合环磷酰胺的免疫抑制治疗并应用血浆置换降低抑制物滴度。目前尚无针对AFVI治疗方案的指南共识，相关治疗剂量可参考Ⅷ/Ⅸ因子抑制物诊疗指南，或根据患者治疗反应及参考相关文献进行调整。在本例患者中，由于患者同时存在门静脉炎，我们沿用了前期甲泼尼龙方案，并加用环磷酰胺。目前建议血浆置换量为1～1.5倍血浆总量，但受限于血浆的可获得性，我们将首次上机的置换总量设为1.2倍血浆总量。患者在接受血浆置换后凝血功能可明显改善，但往往随着抑制物的再次生成而出现反跳。本例患者共接受3次血浆置换，直至免疫抑制治疗起效，凝血功能处于稳步恢复期后停止血浆置换治疗。

AFVI的预后与其诱发病因相关，其中与药物相关的病例预后最佳，大部分患者血清抑制物能在治疗后消失，甚至可自然消退。尽早去除诱发因素对预后至关重要。有报道表明，AFVI的死亡率约为14％，然而这些患者的死亡与其基础疾病及临床情况关系更大，而非AFVI本身[3]。本例患者在针对性治疗1个月后凝血功能恢复至正常范围，且随访3个月凝血功能仍在正常范围，由于患者拒绝复查抑制物滴度，无法明确抑制物消退时间点及消退速度。
